# Interactions between Sterically Stabilized Nanoparticles: The Effects of Brush Bidispersity and Chain Stiffness

**DOI:** 10.3390/polym13142296

**Published:** 2021-07-13

**Authors:** Sergei A. Egorov

**Affiliations:** Department of Chemistry, University of Virginia, Charlottesville, VA 22901, USA; sae6z@virginia.edu

**Keywords:** polymer brushes, density functional theory, simulation, semiflexible polymers

## Abstract

Density Functional Theory is employed to study structural properties and interactions between solvent-free polymer-grafted nanoparticles. Both monodisperse and bidisperse polymer brushes with variable chain stiffness are considered. The three major control parameters are the grafting density, the grafted chain length, and its stiffness. The effect of these parameters on the brush-brush overlap and attractive interaction strength is analyzed. The Density Functional Theory results are compared with the available simulation data, and good quantitative agreement is found.

## 1. Introduction

Colloids and nanoparticles grafted with polymer chains play an important role in the scientific and technological fields of colloid stabilization [1,2], lubrication [3], and adhesion [4]. As such, these systems have received significant experimental [5,6] and theoretical [7,8] attention. One way of controlling the properties of sterically stabilized systems is by tuning either the solvent quality [9,10,11,12] or the properties of the (ungrafted) polymer matrix [13,14]. However, this method does not apply to the technologically important solvent-free (matrix-free) nanocomposites consisting of inorganic cores grafted with polymeric brushes [15]. These nanoparticle-organic hybrid materials exhibit superior thermal and mechanical properties [16], and hold a strong promise for various advanced applications [17,18]. For example, brush coating technology is used for the alignment of one-dimension nanomaterials [19]. Accordingly, these materials have been actively studied both experimentally [20,21,22] and theoretically [15,21,23,24,25].

Of particular importance for the present work is the recent Molecular Dynamics (MD) simulation study of solvent-free polymer brushes, where detailed results are reported on the equilibrium density profiles and the brush overlap as a function of the grafting density and grafted chain length [26]. In addition to monodisperse brushes, equimolar bidisperse brushes were also considered, which is important due to the role of bimodal surface ligands in tunability of nanocomposites [27,28]. While MD simulations provide exact results (with inevitable statistical noise) for a given microscopic model, they can also be time-consuming for systems involving long polymer chains and high grafting densities [8]. An appealing alternative is provided by mean-field techniques, such as self-consistent field theory [10,29,30], integral equation theory [31,32,33,34], and density functional theory (DFT) [9,12,35]. The latter method has been already applied to study solvent-free polymer brushes [36]. However, to the best of our knowledge, the existing studies of solvent-free brush systems are limited to fully flexible grafted chains. At the same time, MD simulations indicate that the chain stiffness plays an important role in controlling the interactions between polymer-grafted nanoparticles in a polymer matrix [37], as well as the mechanical properties of polymer nanocomposites [38]. Hence, one can expect the stiffness to be a useful control parameter in the matrix-free case as well [39].

The central goal of the present work is to develop a DFT approach for the particles grafted with chains of variable stiffness in order to study their structure and interactions. The three main control parameters to be considered are the grafting density, the grafted chain length, and its stiffness. For the fully flexible brushes, the DFT accuracy will be assessed via a detailed comparison of the structural results with the available simulation data, both for monodisperse and bidisperse cases.

The outline of the remainder of the paper is as follows. In Section 2 we specify our microscopic model and in Section 3 we outline the theoretical methods used in the present study to compute the structural and energetic properties of the polymer nanocomposites. Section 4.1 and Section 4.2 presents our results for flexible and semiflexible monodisperse brushes, respectively, and the corresponding results for bidisperse brushes are given in Section 4.3 and Section 4.4. Section 5 concludes the paper.

## 2. Microscopic Model

In order to be able to compare our theoretical results directly with MD simulations, we employ a microscopic model that resembles very closely the model used in the recent simulation study [26]. Specifically, a monodisperse polymer brush is modeled as a flat structureless wall (located in xy-plane) which is grafted uniformly with polymer chains of length *N* at grafting density $\sigma_{g}$. In the case of a bidisperse brush, an equimolar mixture of 2 polymers of length $N_{1}$ and $N_{2}$ is grafted to the wall at the total grafting density $\sigma_{g}$. The monomers comprising the chain are spheres of diameter σ, and all the bond lengths are fixed at $l_{b}=\sigma$ (σ will be used as length unit throughout this work). In order to study the effect of chain flexibility on the brush structural properties we employ a bond-bending potential [40,41]:

(1) $$V_{\mathrm{bend}}\left(\theta_{ijk}\right)=\epsilon_{b}\left[1-\cos\left(\theta_{ijk}\right)\right]=\epsilon_{b}\left[1-\frac{s_{i}\cdot s_{i+1}}{\sigma^{2}}\right],$$

where $\theta_{ijk}$ is the bond angle formed between the two subsequent vectors $s_{i}$ and $s_{i+1}$ along the bonds connecting monomers i,j=i+1 and j,k=i+2, i.e., $s_{i}=r_{i+1}-r_{i}$ and $s_{i+1}=r_{i+2}-r_{i+1}$. The energy parameter $\epsilon_{b}$ then controls the persistence length $l_{p}$, which is defined as [42]

(2) $$l_{p}/l_{b}=-1/\ln\langle \cos\theta_{ijk}\rangle .$$

While for flexible polymers ($\epsilon_{b}=0$ in Equation (1)) one has the persistence length $l_{p}\approx l_{b}$, for semiflexible chains with $\epsilon_{b}\geq 2$ one has the persistence length $l_{p}/l_{b}\approx \epsilon_{b}/k_{B}T=\kappa$, where κ is the dimensionless stiffness parameter [40,41].

The interactions between non-bonded monomers are described via Lennard-Jones potential, truncated and shifted at $r_{\mathrm{cut}}=2.5\sigma$:

(3) $$\begin{array}{c} U_{pp}\left(r\right)=\left\{\begin{array}{cc} 4\epsilon [{\left(\frac{\sigma }{r}\right)}^{12}-{\left(\frac{\sigma }{r}\right)}^{6}-{\left(\frac{\sigma }{r_{\mathrm{cut}}}\right)}^{12}+{\left(\frac{\sigma }{r_{\mathrm{cut}}}\right)}^{6}], & r<r_{\mathrm{cut}}\\ 0, & r\geq r_{\mathrm{cut}} \end{array}\right. \end{array}$$

where ϵ is the potential well depth. The latter will be used as the energy unit throughout this work, with the temperature fixed at $T^{*}=k_{B}T/\epsilon =1.0$.

Finally, the interaction between the monomers and the wall is modeled via the following potential [26]:

(4) $$\begin{array}{c} U_{wp}\left(z\right)=\left\{\begin{array}{cc} 4\epsilon [{\left(\frac{\sigma }{z}\right)}^{12}-{\left(\frac{\sigma }{z}\right)}^{6}+\frac{1}{4}], & z<2^{1/6}\sigma \\ 0, & z\geq 2^{1/6}\sigma , \end{array}\right. \end{array}$$

where *z* is the distance of the monomer from the wall.

## 3. Density Functional Theory

As a starting point of any DFT-based treatment, [43,44] one writes an expression of the grand free energy, Ω, as a functional of the polymer density profile $\rho_{p}\left(R_{p}\right)$, where $R_{p}=\left(r_{1},r_{2},\cdots ,r_{N}\right)$ is a collective variable with the individual monomer coordinates $r_{i}$. The minimization of Ω with respect to $\rho_{p}\left(R_{p}\right)$ yields the equilibrium polymer density distribution. The functional Ω is related to the Helmholtz free energy functional, *F*, via a Legendre transform:

(5) $$\Omega \left[\rho_{p}\left(R_{p}\right)\right]=F\left[\rho_{p}\left(R_{p}\right)\right]+\int dR_{p}\rho_{p}\left(R_{p}\right))\left[V_{ext}\left(R_{p}\right)-\mu \right],$$

where μ is the polymer chemical potential (which will be set to 0 throughout this study due to the fact that the chains are grafted to the wall at a fixed grafting density), and $V_{ext}\left(R_{p}\right)$ is the external field, which in the present case is due to the interaction of the polymer beads with the wall:

(6) $$V_{ext}\left(R_{p}\right)=\sum_{i=1}^{N}U_{wp}\left(r_{i}\right).$$

We employ the following approximation for the Helmholtz free energy functional, which separates it into ideal and excess parts according to: [45]

(7) $$F\left[\rho_{p}\left(R_{p}\right)\right]=F_{\mathrm{id}}\left[\rho_{p}\left(R_{p}\right)\right]+F_{\mathrm{ex}}\left[\rho \left(r\right)\right],$$

with the ideal functional given by [46,47]:

(8) $$\beta F_{\mathrm{id}}\left[\rho_{p}\left(R_{p}\right)\right]=\int dR_{p}\rho_{p}\left(R_{p}\right))\left[\ln\rho_{p}\left(R_{p}\right)-1\right]+\beta \int dR_{p}\rho_{p}\left(R_{p}\right)V_{b}\left(R_{p}\right)+\beta \sum_{i=1}^{N-2}\int dR_{p}\rho_{p}\left(R_{p}\right)V_{\mathrm{bend}}\left(s_{i},s_{i+1}\right),$$

where $\beta =1/(k_{B}T)$, $V_{\mathrm{bend}}$ is given by Equation (1), and $V_{b}\left(R_{p}\right)$ is the binding energy given by [48]:

(9) $$\exp\left[-\beta V_{b}\left(R_{p}\right)\right]=\prod_{i=1}^{N-1}\frac{\delta (|r_{i}-r_{i+1}|-\sigma )}{4\pi \sigma^{2}}=\prod_{i=1}^{N-1}g_{b}\left(|r_{i}-r_{i+1}\right).$$

Additionally, the innermost (i=1) bead of each chain is tethered to the wall via a grafting potential $\exp\left[-\beta v_{g}\left(r_{1}\right)\right]=\delta \left(z_{1}-z_{g}\right)$, where $z_{g}$ is the position of the wall (located in xy-plane) along the *z*-axis.

The excess term is written as a functional of the monomer density given by: [46,47]

(10) $$\rho \left(r\right)=\int dR_{p}\sum_{i=1}^{N}\delta \left(r-r_{i}\right)\rho_{p}\left(R_{p}\right).$$

We write the excess free energy functional as a sum of repulsive and attractive terms [45]:

(11) $$F_{\mathrm{ex}}\left[\rho \left(r\right)\right]=F_{\mathrm{rep}}\left[\rho \left(r\right)\right]+F_{\mathrm{att}}\left[\rho \left(r\right)\right].$$

For the former, we adopt the weighted density approximation [49]:

(12) $$\beta F_{\mathrm{rep}}\left[\rho \left(r\right)\right]=\int dr\rho \left(r\right)f_{\mathrm{rep}}\left(\bar{\rho }\left(r\right)\right),$$

with the weighted density given by:

(13) $$\bar{\rho }\left(r\right)=\int dr^{\prime }\rho \left(r^{\prime }\right)w(|r-r^{\prime }\left|\right).$$

In the above, $f_{\mathrm{rep}}\left(\rho \right)$ is the excess free energy density per site of the polymer melt with site density ρ arising from the short-ranged hard-core repulsive interactions. We compute it from the Wertheim’s expression which was obtained on the basis of the first-order thermodynamic perturbation theory [50]:

(14) $$f_{\mathrm{rep}}\left(\rho \right)=\frac{4\eta -3\eta^{2}}{{\left(1-\eta \right)}^{2}}-\left(1-\frac{1}{N}\right)\ln\frac{1-\eta /2}{{\left(1-\eta \right)}^{3}}$$

where $\eta =\pi \sigma^{3}\rho /6$ is the monomer packing fraction.

In the present work we employ the simple square-well form for the weighting function w(r), whose range is given by the diameter σ of the polymer segment [12]:

(15) $$w\left(r\right)=\frac{3}{4\pi \sigma^{3}}\Theta \left(\sigma -r\right),$$

where Θ(r) is the Heaviside step function. While more sophisticated forms of the weighting function are available in the literature (e.g., those used in the Fundamental Measure Theory version of DFT [51]), earlier studies [52] have shown relative insensitivity of DFT results for polymeric systems to the specific choice of the weight function.

Regarding the attractive contribution to the excess free energy, Ref. [53] in our earlier DFT study of nanoparticle interactions in a polymer melt, Ref. [54] we have found that the most accurate results for polymer density profiles and PMFs were obtained using a simple mean-field approximation for the attractive part of $F_{\mathrm{ex}}$:

(16) $$F_{\mathrm{att}}\left[\rho \left(r\right)\right]=\frac{1}{2}\int dr\int dr^{\prime }\rho \left(r\right)\rho \left(r^{\prime }\right)U_{pp}^{\mathrm{att}}(|r-r^{\prime }\left|\right),$$

where [55]

(17) $$\begin{array}{c} U_{pp}^{\mathrm{att}}\left(r\right)=\left\{\begin{array}{cc} U_{pp}\left(2^{1/6}\sigma \right), & r\leq 2^{1/6}\sigma \\ U_{pp}\left(r\right), & 2^{1/6}\sigma <r\leq r_{cut}\\ 0, & r>r_{cut}. \end{array}\right. \end{array}$$

The minimization of the grand free energy functional Ω yields the following result for the equilibrium polymer density profile [56]:

(18) $$\rho_{p}\left(R_{p}\right)=N_{p}\delta \left(z_{1}-z_{g}\right)\prod_{i=1}^{N-1}g_{b}\left(|r_{i}-r_{i+1}\right)\prod_{i=1}^{N-2}\exp\left[-\beta V_{\mathrm{bend}}\left(s_{i},s_{i+1}\right)\right]\prod_{i=1}^{N}\exp\left[-\lambda \left(r_{i}\right)\right],$$

where

(19) $$\lambda \left(r\right)=\beta \frac{\delta F_{ex}}{\delta \rho (r)}+\beta U_{wp}\left(r\right),$$

and $N_{p}$ is the normalization constant chosen to give the desired grafting density $\sigma_{g}$. Substitution of $\rho_{p}\left(R_{p}\right)$ into Equation (10) then yields an integral equation for the monomer density distribution ρ(r) which needs to be solved numerically [56].

In this study we focus on two opposing interacting polymer brushes with the two parallel walls located at $z_{g1}$ and $z_{g2}$, respectively. In this geometry the equilibrium monomer density distribution ρ(z) is a function of a single variable *z*. The potential of mean force (PMF) W(z) between the two brushes as a function of the distance between the walls is obtained by taking the difference between the free energy *F* at the wall separation *z* and at the wall separation $z_{max}=2N\sigma$, where the two bushes no longer overlap [57]. The value z=H where W(z) passes through a minimum corresponds to the equilibrium separation between the two walls. Following the MD simulation study [26], we define the overlap parameter *P* between the two brushes at the equilibrium separation as follows:

(20) $$P=\frac{\int_{z_{g1}}^{z_{g2}}dz\left[\rho_{1}\left(z\right)\cap \rho_{2}\left(z\right)\right]}{\int_{z_{g1}}^{z_{g2}}dz\left(\rho_{1}\left(z\right)+\rho_{2}\left(z\right)\right)},$$

where $\rho_{1}\left(z\right)$ and $\rho_{2}\left(z\right)$ are the monomer density distributions of the two brushes grafted at $z_{g1}$ and $z_{g2}$, respectively.

We note that our description of the DFT methodology has been formulated for monodisperse brushes comprised of grafted chains of length *N*. In what follows, we will also consider bidisperse brushes comprised of two types of grafted chains of length $N_{1}$ and $N_{2}$, respectively (all other properties of these two chain types will be taken to be identical, e.g., their stiffness parameter $\kappa_{1}=\kappa_{2}$). The above DFT formalism can be generalized to the case of a two-component system in a straightforward way [58,59].

Regarding the numerical implementation of the DFT procedure, the integral equation for the monomer density distribution ρ(z) was solved numerically on an equidistant grid with the grid spacing Δz=0.02. Simple Picard iteration procedure was employed [60], and tolerance criterion for terminating the iterative procedure was set to $10^{-6}$.

## 4. Results

We employ the DFT formalism outlined in Section 3 to study both structural (overlap *P* given by Equation (20)) and energetic (PMF W(z)) properties of two overlapping flat brushes. Both monodisperse and bidisperse brushes are considered, with three main control parameters being the grafted chain length *N*, the grafted chain stiffness parameter κ, and the grafting density $\sigma_{g}$.

### 4.1. Monodisperse Brush: Flexible Chains

**Figure 1:**
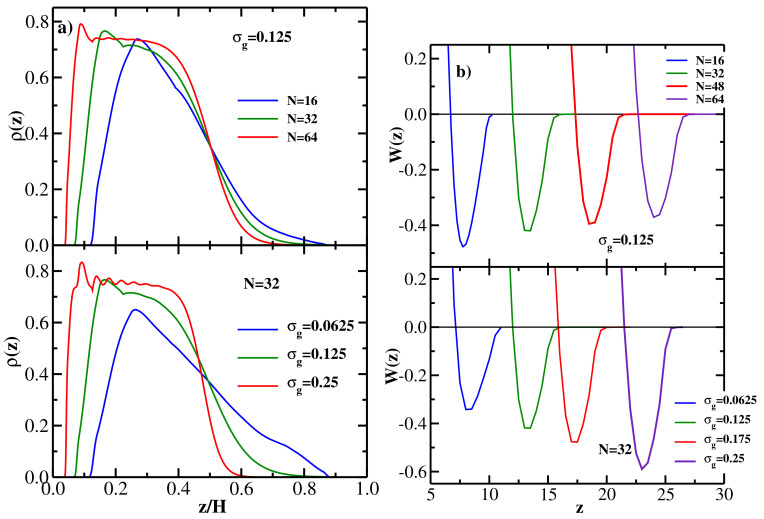
(**a**) Upper panel: DFT results for the equilibrium monomer density distributions for monodisperse flexible brushes at equilibrium separation *H* vs z/H; grafting density is $\sigma_{g}=0.125$ and three values of the grafted chain length are considered: N=16, 32, and 64. Only monomer density distributions of the chains grafted at the left wall are shown; the distributions of the chains grafted at the right wall are mirror-symmetric (around the mid-point of the gap between the walls) with respect to the left-wall grafted chains. Lower panel: DFT results for the equilibrium monomer density distributions for monodisperse flexible brushes at equilibrium separation *H* vs z/H; grafted chain length is N=32 and three values of grafting density are considered: $\sigma_{g}=0.0625$, $\sigma_{g}=0.125$, and $\sigma_{g}=0.25$. (**b**) Upper panel: PMF between two monodisperse flexible brushes as a function of wall-wall separation at the grafting density $\sigma_{g}=0.125$ for four values of the grafted chain length *N*, as indicated. Lower panel: PMF between two monodisperse flexible brushes as a function of wall-wall separation for the grafted chain length N=32 for four values of the grafting density $\sigma_{g}$, as indicated.

We start by studying monodisperse brushes comprised of fully flexible (κ=0) chains of length *N*. Figure 1a shows representative DFT results for the grafted chain monomer density distributions at the equilibrium wall-wall separation *H*. Only the density profiles ρ(z) of the monomers of the chains grafted at the left wall are shown (as a function of z/H), the corresponding profiles of the chains grafted at the right wall are mirror symmetric (around the mid-point of the gap between the two walls), because the two opposing brushes are identical. The upper panel of Figure 1a shows the DFT results for the grafting density $\sigma_{g}=0.125$ and three values of the grafted chain length: N=16, 32, and 64. The lower panel of Figure 1a shows the corresponding results for the grafted chain length N=32 and three values of the grafting density: $\sigma_{g}=0.0625$, $\sigma_{g}=0.125$, and $\sigma_{g}=0.25$.

**Figure 2:**
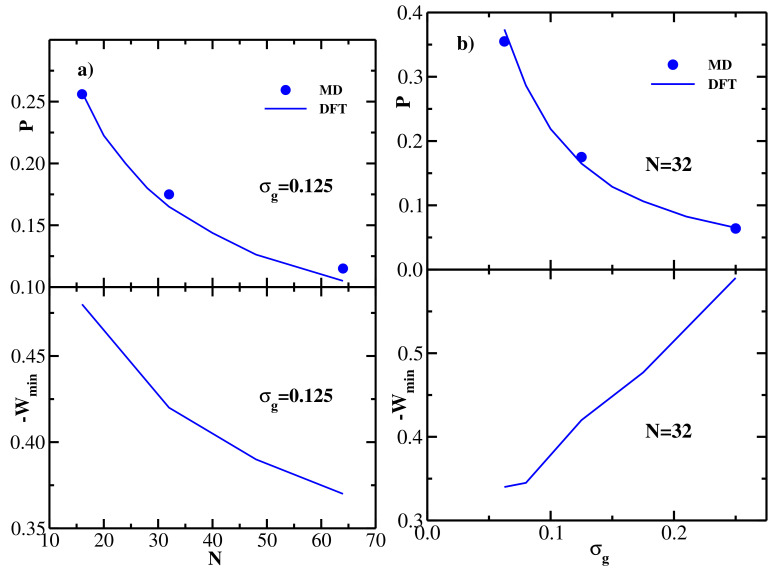
(**a**) Upper panel: overlap *P* defined via Equation (20) as a function of the grafted chain length *N* for two monodisperse flexible brushes at equilibrium separation; grafting density is $\sigma_{g}=0.125$. Solid line shows the present DFT results, and symbols are from MD simulations [26]. Lower panel: DFT results for the (negative) minimum of the PMF between two monodisperse flexible brushes as a function of the grafted chain length *N*; grafting density is $\sigma_{g}=0.125$. (**b**) Upper panel: overlap *P* defined via Equation (20) as a function of the grafting density $\sigma_{g}$ for two monodisperse flexible brushes at equilibrium separation; grafted chain length is N=32. Solid line shows the present DFT results, and symbols are from MD simulations [26]. Lower panel: DFT results for the (negative) minimum of the PMF between the two monodisperse flexible brushes as a function of the grafting density $\sigma_{g}$; grafted chain length is N=32.

One sees from Figure 1a that both with increasing chain length (at a fixed grafting density, upper panel) and with increasing grafting density (at a fixed chain length, lower panel), the monomer density distribution becomes more bell-shaped (i.e., more localized at the grafting wall), in agreement with MD simulation results [26]. Keeping in mind that the two brushes grafted at the left and right walls are identical (and their respective density profiles are mirror symmetric around the gap mid-point), it follows that the overlap between the two opposing brushes decreases both with increasing *N* (at fixed $\sigma_{g}$) and with increasing $\sigma_{g}$ (at fixed *N*). These trends are also in agreement with MD simulation results [26], and can be quantified via the overlap parameter *P* given by Equation (20) (see the discussion of Figure 2 below).

Moving next to the PMF between the two brushes, the upper panel of Figure 1b shows DFT results for W(z) for 4 different chain lengths at the fixed grafting density $\sigma_{g}=0.125$, while the lower panel gives DFT results for W(z) for 4 different grafting densities at the fixed grafted chain length N=32. The upper panel shows that the attractive interaction between the two brushes becomes weaker with increasing chain length (at fixed $\sigma_{g}$), while the lower panel indicates the the attraction between the two brushes becomes stronger with increasing grafting density (at fixed *N*). In addition, the equilibrium wall-wall separation (corresponding to the minimum location of W(z)) increases monotonically both with *N* and with $\sigma_{g}$, as one would expect [10].

The DFT results for both structural (overlap) and energetic (PMF) properties of monodisperse flexible brushes and their dependence on the chain length and grafting density are summarized in Figure 2, where a quantitative comparison with the corresponding MD data [26] is also performed. In particular, the upper panel of Figure 2a shows the MD [26] (symbols) and DFT (lines) results for the overlap *P* as a function of the grafted chain length *N*; the grafting density is fixed at $\sigma_{g}=0.125$. The overlap is computed at the equilibrium separation between the two brushes z=H corresponding to the minimum of the brush-brush PMF (see the upper panel of Figure 1b). One sees that DFT is in good quantitative agreement with MD, both methods show that the overlap decreases monotonically with the grafted chain length *N*, as one could already anticipate from the density profiles shown in the upper panel of Figure 1a. The lower panel of Figure 2a shows the (negative) minimum of the PMF between the two brushes as a function of the grafted chain length *N*, −$W_{\min}$ decreases monotonically with *N*, indicating that the attraction between the two bushes becomes weaker as the chain length is increased (see the upper panel of Figure 1b).

The upper panel of Figure 2b shows the MD [26] (symbols) and DFT (lines) results for the overlap *P* as a function of the grafting density $\sigma_{g}$, the grafted chain length is fixed at N=32. DFT is again seen to be in good agreement with MD, both methods show that the overlap decreases monotonically with the grafting density, thereby quantifying the qualitative trend seen in the density profiles shown in the lower panel of Figure 1a. The lower panel of Figure 2b shows the (negative) minimum of the PMF between the two brushes as a function of the grafting density, -$W_{\min}$ increases monotonically with $\sigma_{g}$, indicating that the attraction between the two bushes becomes stronger as the grafting density is increased (see the lower panel of Figure 1b).

In summary, with increasing chain length at fixed grafting density (Figure 2a), both *P* and $-W_{\min}$ decrease, i.e., the amount of overlap directly correlates with the strength of the attractive brush-brush interaction. At the same time, increasing the grafting density at fixed *N* (Figure 2b) leads to decreasing *P*, while $-W_{\min}$ increases. In other words, in the latter case the amount of overlap anti-correlates with the strength of the attractive brush-brush interaction.

### 4.2. Monodisperse Brush: Semiflexible Chains

**Figure 3:**
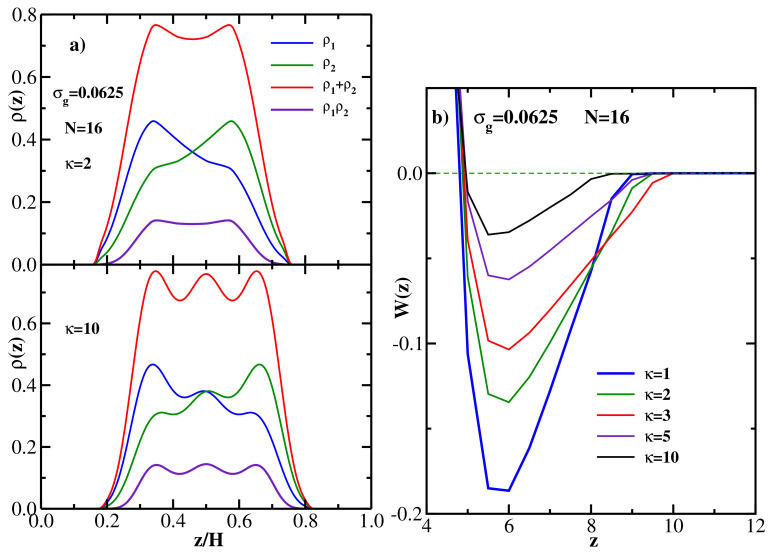
(**a**) Upper panel: DFT results for the equilibrium monomer density distributions for two monodisperse semiflexible brushes at equilibrium separation *H* vs z/H; grafting density is $\sigma_{g}=0.0625$, grafted chain length is N=16, and the chain stiffness parameter is κ=2. $\rho_{1}\left(z\right)$ is the monomer density distribution of the brush grafted at the left wall ($z_{g1}=0$), $\rho_{2}\left(z\right)$ is the monomer density distribution of the brush grafted at the right wall ($z_{g2}=H$). The sum $\rho_{1}\left(z\right)+\rho_{2}\left(z\right)$ and the product $\rho_{1}\left(z\right)\rho_{2}\left(z\right)$ of the two profiles are also shown. Lower panel: same as the upper panel but for the chain stiffness parameter κ=10. (**b**) PMF between two monodisperse semiflexible brushes as a function of wall-wall separation at the grafting density $\sigma_{g}=0.0625$ and the grafted chain length N=16 for five values of the grafted chain stiffness parameter κ, as indicated.

**Figure 4:**
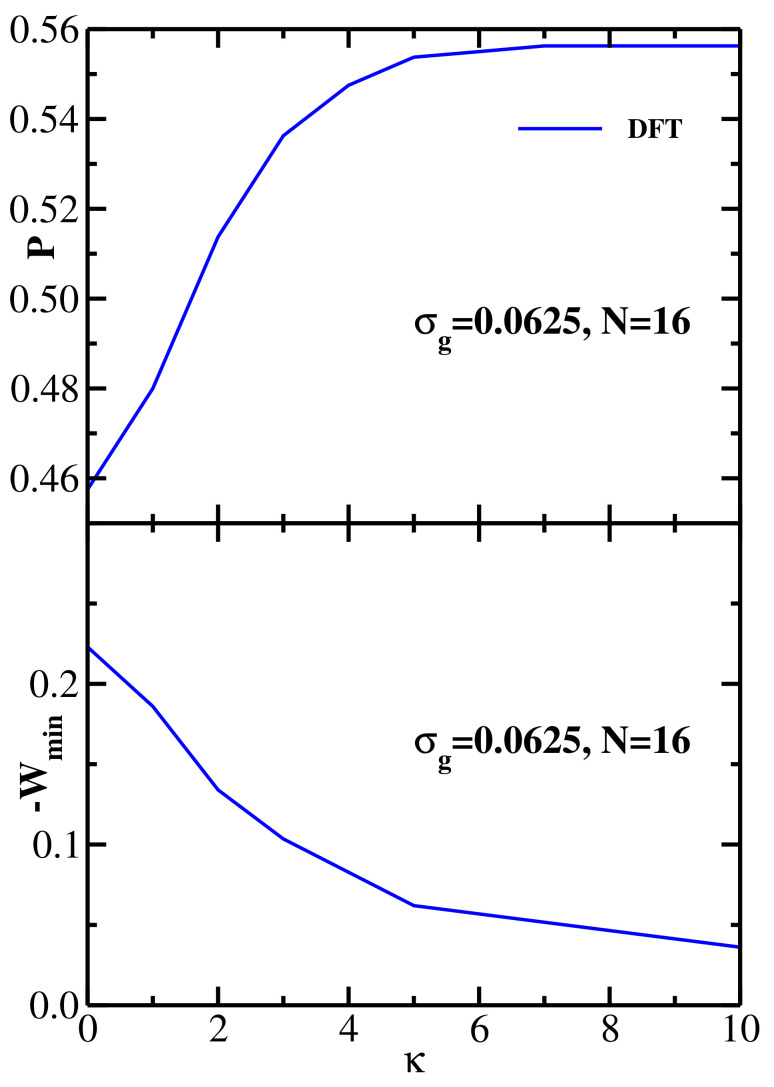
Upper panel: DFT results for the overlap *P* as a function of the grafted chain stiffness parameter κ for two monodisperse semiflexible brushes at equilibrium separation *H*; grafting density is $\sigma_{g}=0.0625$ and grafted chain length is N=16. Lower panel: DFT results for the (negative) minimum of the PMF between two monodisperse semiflexible brushes as a function of the grafted chain stiffness parameter κ.

Having considered fully flexible brushes, we next study the effect of varying the grafted chain stiffness on the structural and energetic properties of monodisperse brushes. To this end, we fix the grafting density at $\sigma_{g}=0.0625$ and the grafted chain length at N=16, and vary its stiffness parameter κ. Representative DFT results for the monomer density distribution of two opposing monodisperse semiflexible brushes (at equilibrium separation *H*) are shown in Figure 3a. The upper panel of Figure 3a presents the results for ρ(z) vs z/H for κ=2, while the lower panel gives the results for stiffer chains with κ=10. Here $\rho_{1}\left(z\right)$ is the monomer density distribution of the brush grafted at the left wall ($z_{g1}=0$), and $\rho_{2}\left(z\right)$ is the (mirror-symmetric) monomer density distribution of the brush grafted at the right wall ($z_{g2}=H$). The sum $\rho_{1}\left(z\right)+\rho_{2}\left(z\right)$ and the product $\rho_{1}\left(z\right)\rho_{2}\left(z\right)$ of the two profiles are also shown. The width at half-maximum of the latter serves as a measure of the thickness of the interpenetration region [61]. The latter quantity can be viewed as an alternative to overlap *P* in characterizing the degree of interpenetration of the two brushes. Both these measures show that the overlap is stronger in the lower panel compared to the upper panel, indicating that the overlap between the brushes increases with increasing chain stiffness (see also the upper panel of Figure 4 below). This trend is due to the fact that the extension of the chains away from the grafting wall increases with their stiffness.

Considering next the PMF between the two monodisperse semiflexible brushes, Figure 3b shows the DFT results for W(z) for 5 different values of the chain stiffness parameter at the fixed grafting density $\sigma_{g}=0.0625$ and chain length N=16. DFT results show that the strength of the brush-brush attraction decreases with increasing chain stiffness. This general trend is in general qualitative agreement with MD simulation results [37], but no quantitative comparison between MD and DFT is possible due to the fact that MD simulations were performed for spherical brushes under athermal conditions. One can also note that the equilibrium wall-wall separation decreases slightly with increasing chain stiffness, albeit the effect is rather weak.

The DFT results for both structural and energetic properties of monodisperse semiflexible brushes and their dependence on the chain stiffness are summarized in Figure 4, with the upper panel showing the overlap *P* and the lower panel presenting the (negative) PMF minimum −$W_{\min}$ as a function of the chain stiffness parameter. As expected from the trends seen in Figure 3, *P* increases and −$W_{\min}$ decreases monotonically with κ, with the former reaching saturation around κ=10 (for longer chains one would expect the saturation to occur at larger values of κ [62]). As in the case of Figure 2b, the amount of overlap anticorrelates with the attraction strength between the two brushes.

To summarize the DFT results for monodisperse brushes, one can identify 3 control parameters for tuning the brush-brush overlap and the attraction strength: the grafting density, the grafted chain length, and the chain stiffness. On the one hand, the overlap decreases monotonically with $\sigma_{g}$ and *N*, but increases with κ. On the other hand, the attraction strength decreases monotonically with *N* and κ, but increases with $\sigma_{g}$.

### 4.3. Bidisperse Brush: Flexible Chains

Motivated by the MD results presented in Ref. [26], we now consider bidisperse flexible brushes. In order to perform a direct comparison between MD and DFT results, we restrict our attention to the equimolar case studied by MD [26], whereby the mole fractions of both components are equal to 0.5, and the two components only differ in their chain lengths, $N_{1}$ and $N_{2}$ respectively.

**Figure 5:**
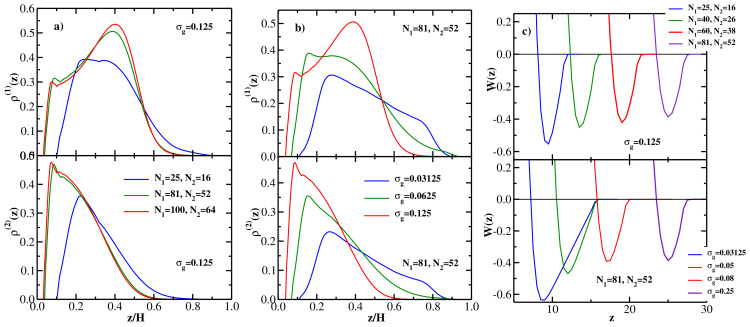
(**a**) Upper panel: DFT results for the equilibrium monomer density distributions for equimolar bidisperse flexible brushes at equilibrium separation *H* vs z/H; grafting density is $\sigma_{g}=0.125$ and three pairs of grafted chain lengths are considered: ($N_{1}=25$, $N_{2}=16$), ($N_{1}=81$, $N_{2}=52$), and ($N_{1}=100$, $N_{2}=64$). Only monomer density distributions of the longer chains (length $N_{1}$) grafted at the left wall are shown; the distributions of the chains grafted at the right wall are mirror-symmetric (around the mid-point of the gap between the walls) with respect to the left-wall grafted chains. Lower panel: same as the upper panel but for the shorter grafted chains (length $N_{2}$). (**b**) Upper panel: DFT results for the equilibrium monomer density distributions for equimolar bidisperse flexible brushes at equilibrium separation *H* vs z/H; grafted chain lengths are ($N_{1}=81$, $N_{2}=52$) and three values of the grafting density are considered: $\sigma_{g}=0.03125$, $\sigma_{g}=0.0625$, and $\sigma_{g}=0.125$. Lower panel: same as the upper panel but for the shorter grafted chains (length $N_{2}$). (**c**) Upper panel: PMF between two equimolar bidisperse flexible brushes as a function of wall-wall separation at the grafting density $\sigma_{g}=0.125$ for four pairs of the grafted chain lengths ($N_{1}$, $N_{2}$), as indicated. Lower panel: PMF between two equimolar bidisperse flexible brushes as a function of wall-wall separation for the grafted chain lengths ($N_{1}=81$, $N_{2}=52$) for four values of the grafting density $\sigma_{g}$, as indicated.

**Figure 6:**
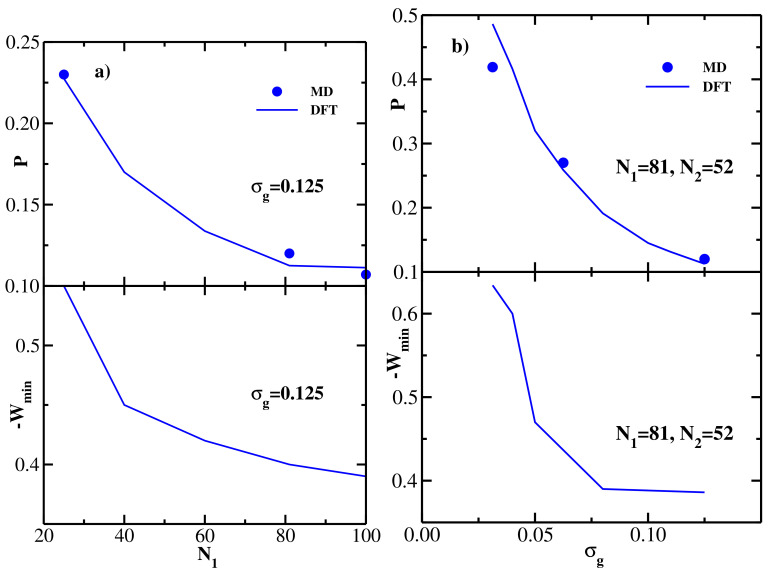
(**a**) Upper panel: overlap *P* defined via Equation (20) as a function of the longer grafted chain length $N_{1}$ for two equimolar bidisperse flexible brushes at equilibrium separation; grafting density is $\sigma_{g}=0.125$, and the shorter grafted chain length $N_{2}$ is given by the integer part of the value $16N_{1}/25$. Solid line shows the present DFT results, and symbols are from MD simulations [26]. Lower panel: DFT results for the (negative) minimum of the PMF between two equimolar bidisperse flexible brushes as a function of the longer grafted chain length $N_{1}$; grafting density is $\sigma_{g}=0.125$, and the shorter grafted chain length $N_{2}$ is given by the integer part of the value $16N_{1}/25$. (**b**) Upper panel: overlap *P* defined via Equation (20) as a function of the grafting density $\sigma_{g}$ for two equimolar bidisperse flexible brushes at equilibrium separation; grafted chain lengths are $N_{1}=81$ and $N_{1}=52$. Solid line shows the present DFT results, and symbols are from MD simulations [26]. Lower panel: DFT results for the (negative) minimum of the PMF between the two equimolar bidisperse flexible brushes as a function of the grafting density $\sigma_{g}$; grafted chain lengths are $N_{1}=81$ and $N_{2}=52$.

Figure 5a shows the DFT results for the monomer density distributions of bidisperse flexible brushes at equilibrium separation; the grafting density is fixed at $\sigma_{g}=0.125$ and three pairs of grafted chain lengths are considered: ($N_{1}=25$, $N_{2}=16$), ($N_{1}=81$, $N_{2}=52$), and ($N_{1}=100$, $N_{2}=64$) (note that the ratio $N_{1}/N_{2}$ is kept constant at the same value as in the MD study [26]). The upper panel of Figure 5a gives the density profiles of the longer chains ($\rho^{\left(1\right)}\left(z\right)$ vs z/H), and the lower panel shows the density profiles of the shorter chains ($\rho^{\left(2\right)}\left(z\right)$ vs z/H). In both panels, only monomer density distributions of the chains grafted at the left wall are shown; the brush grafted at the right wall is identical and its density profile is mirror-symmetric. Keeping this in mind, one sees that with increasing $N_{1}$ and $N_{2}$ the overlap between the two brushes decreases, in agreement with MD results [26] (see also the discussion of Figure 6a below). In addition, for the shortest pair considered here ($N_{1}=25$, $N_{2}=16$), the density profiles of both components are rather similar (with the longer one being more bell-shaped than the shorter one). At the same time, for the other two pairs the shorter component is clearly localized closer to the grafting wall, while the longer one is stretched away from it, again in agreement with simulations [26].

Figure 5b shows the DFT results for the monomer density distributions of bidisperse flexible brushes at equilibrium separation; the chain lengths are fixed at ($N_{1}=81$, $N_{2}=52$) and three values of the grafting density are considered: $\sigma_{g}=0.03125$, $\sigma_{g}=0.0625$, and $\sigma_{g}=0.125$. The upper panel of Figure 5b gives $\rho^{\left(1\right)}\left(z\right)$ vs z/H for the longer chains and the lower panel shows $\rho^{\left(2\right)}\left(z\right)$ vs z/H for the shorter chains. In both panels, only monomer density distributions of the chains grafted at the left wall are shown; the brush grafted at the right wall is identical and its density profile is mirror-symmetric. One sees that with increasing grafting density the overlap between the two brushes decreases, in agreement with MD results [26] (see also the discussion of Figure 6b below). Furthermore, the effect of the grafting density on the density profiles and the brush overlap is seen to be much stronger compared to the effect of the chain lengths (Figure 5a). In addition, increasing $\sigma_{g}$ results in the increased localization of the shorter chains at the grafting wall and the stretching of the longer chains away from it. Once again, all the trends predicted by DFT are in good agreement with the MD simulation data [26].

Moving next to the PMF between the two flexible bidisperse brushes, the upper panel of Figure 5c shows DFT results for W(z) for 4 different pairs of chain lengths at the fixed grafting density $\sigma_{g}=0.125$, while the lower panel gives DFT results for W(z) for 4 different grafting densities at the fixed grafted chain lengths ($N_{1}=81$, $N_{2}=52$). Figure 5c shows that the attractive interaction between the two brushes becomes weaker (and the equilibrium separation increases) both with increasing chain lengths at fixed $\sigma_{g}$ and with increasing grafting density at fixed chain lengths. This behavior can be contrasted with monodisperse brushes, where the chain length and the grafting density have the opposite effect on the brush-brush attraction strength (Figure 1b), while having the same effect on the brush overlap (Figure 2).

The DFT results for both structural (overlap) and energetic (PMF well depth) properties of bidisperse flexible brushes and their dependence on the chain lengths and grafting density are summarized in Figure 6, where a quantitative comparison with the corresponding MD data [26] is also performed. In particular, the upper panel of Figure 6a shows the MD [26] (symbols) and DFT (lines) results for the overlap *P* as a function of the longer grafted chain length $N_{1}$; the grafting density is fixed at $\sigma_{g}=0.125$, and the shorter grafted chain length $N_{2}$ is given by the integer part of the value $16N_{1}/25$. The overlap is computed at the equilibrium separation between the two brushes corresponding to the minimum of the brush-brush PMF (see the upper panel of Figure 5c). One sees that DFT results are in good agreement with MD, both methods show that the overlap decreases monotonically with the grafted chain length $N_{1}$, as one could already anticipate from the density profiles shown in Figure 5a. The lower panel of Figure 6a shows the (negative) minimum of the PMF between the two brushes as a function of the longer grafted chain length $N_{1}$, −$W_{\min}$ decreases monotonically with $N_{1}$, indicating that the attraction between the two bushes becomes weaker as the chain length is increased (see the upper panel of Figure 5c).

The upper panel of Figure 6b shows the MD [26] (symbols) and DFT (lines) results for the overlap *P* as a function of the grafting density $\sigma_{g}$, the grafted chain lengths are fixed at $N_{1}=81$ and $N_{2}=52$. DFT is again seen to be in good agreement with MD, both methods show that the overlap decreases monotonically with the grafting density, thereby quantifying the qualitative trend seen in the density profiles shown in the Figure 5b. Furthermore, the decrease of *P* with $\sigma_{g}$ is much stronger (a factor of 4 over the range of $\sigma_{g}$ studied here) compared to its decrease with $N_{1}$ (a factor of 2). The lower panel of Figure 6b shows the (negative) minimum of the PMF between the two brushes as a function of the grafting density, −$W_{\min}$ decreases monotonically with $\sigma_{g}$, indicating that the attraction between the two bushes becomes weaker as the grafting density is increased (see the lower panel of Figure 5c).

In summary, DFT predicts that both increasing the grafting density and increasing the chain lengths diminish the overlap between two bidisperse flexible brushes, as well as the strength of their attraction, which stands in contrast to the case of monodisperse brushes.

### 4.4. Bidisperse Brush: Semiflexible Chains

**Figure 7:**
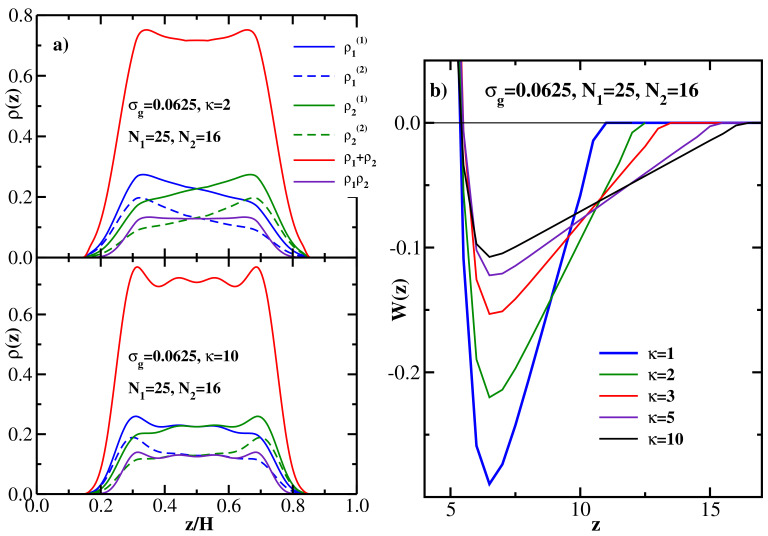
(**a**) Upper panel: DFT results for the equilibrium monomer density distributions for two equimolar bidisperse semiflexible brushes at the equilibrium separation; grafting density is $\sigma_{g}=0.0625$, grafted chain lengths are $N_{1}=25$ and $N_{2}=16$, and the chain stiffness parameter is κ=2. $\rho_{1}^{\left(1\right)}\left(z\right)$ ($\rho_{1}^{\left(2\right)}\left(z\right)$) is the monomer density distribution of the longer (shorter) brush grafted at the left wall ($z_{g1}=0$), $\rho_{2}^{\left(1\right)}\left(z\right)$ ($\rho_{2}^{\left(2\right)}\left(z\right)$) is the monomer density distribution of the longer (shorter) brush grafted at the right wall ($z_{g2}=H$). The sum $\rho_{1}\left(z\right)+\rho_{2}\left(z\right)=\rho_{1}^{\left(1\right)}\left(z\right)+\rho_{1}^{\left(2\right)}\left(z\right)+\rho_{2}^{\left(1\right)}\left(z\right)+\rho_{2}^{\left(2\right)}\left(z\right)$ and the product $\rho_{1}\left(z\right)\rho_{2}\left(z\right)=\left(\rho_{1}^{\left(1\right)}\left(z\right)+\rho_{1}^{\left(2\right)}\left(z\right)\right)\left(\rho_{2}^{\left(1\right)}\left(z\right)+\rho_{2}^{\left(2\right)}\left(z\right)\right)$ of the two total profiles are also shown. Lower panel: DFT results for the equilibrium monomer density distributions for two monodisperse semiflexible brushes at the equilibrium separation; grafting density is $\sigma_{g}=0.0625$, grafted chain lengths are $N_{1}=25$ and $N_{2}=16$, and the chain stiffness parameter is κ=10. (**b**) PMF between two equimolar bidisperse semiflexible brushes as a function of wall-wall separation at the grafting density $\sigma_{g}=0.125$ and the grafted chain lengths $N_{1}=25$ and $N_{2}=16$ for five values of the grafted chain stiffness parameter κ, as indicated.

**Figure 8:**
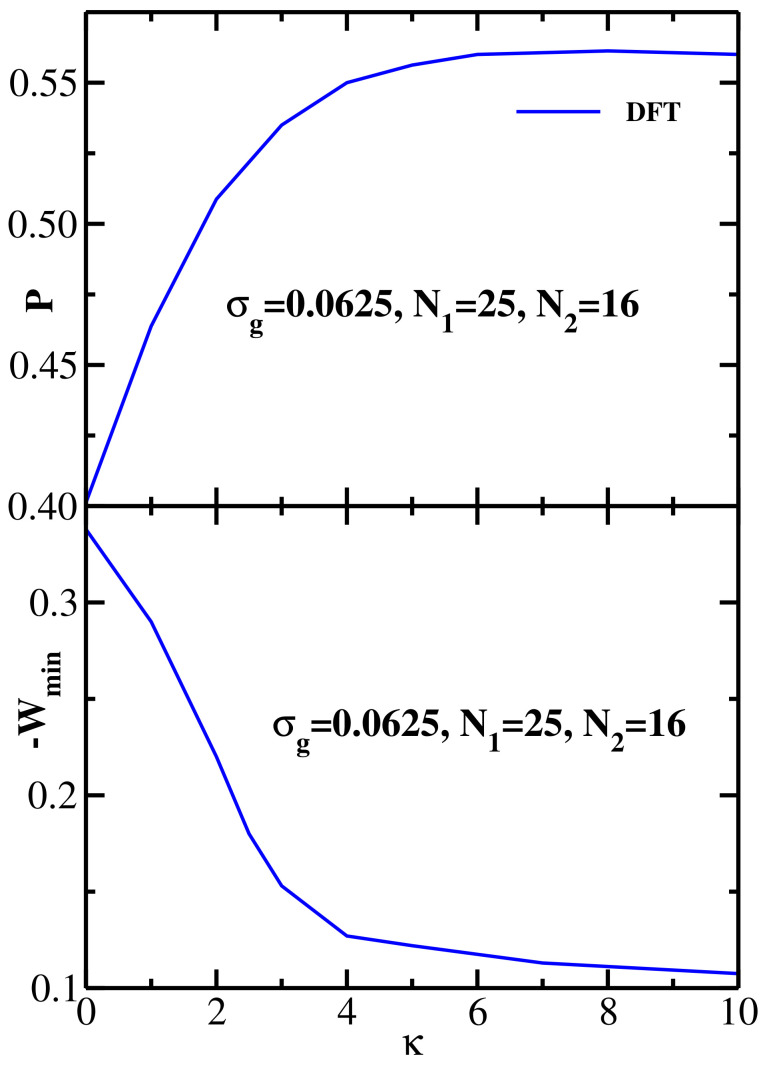
Upper panel: DFT results for the overlap *P* as a function of the grafted chain stiffness parameter κ for two equimolar bidisperse semiflexible brushes at equilibrium separation; grafting density is $\sigma_{g}=0.0625$ and grafted chain lengths are $N_{1}=25$ and $N_{2}=16$. Lower panel: DFT results for the (negative) minimum of the PMF between two equimolar bidisperse semiflexible brushes as a function of the grafted chain stiffness parameter κ; grafting density is $\sigma_{g}=0.0625$ and grafted chain lengths are $N_{1}=25$ and $N_{2}=16$.

In this section we study the effect of varying the grafted chain stiffness on the structural and energetic properties of equimolar bidisperse brushes. Here we fix the grafting density at $\sigma_{g}=0.0625$ and the grafted chain lengths at ($N_{1}=25$, $N_{2}=16$), and simultaneously vary the stiffness parameter of both chains, $\kappa_{1}=\kappa_{2}=\kappa$. Representative DFT results for the monomer density distribution of two opposing bidisperse equimolar semiflexible brushes (at the equilibrium separation *H*) are shown in Figure 7a. The upper panel of Figure 7a presents the results for ρ(z) vs z/H for κ=2, while the lower panel gives the results for stiffer chains with κ=10. Here $\rho_{1}^{\left(1\right)}\left(z\right)$ ($\rho_{1}^{\left(2\right)}\left(z\right)$) is the monomer density distribution of the longer (shorter) brush grafted at the left wall ($z_{g1}=0$), while $\rho_{2}^{\left(1\right)}\left(z\right)$ ($\rho_{2}^{\left(2\right)}\left(z\right)$) is the mirror-symmetric monomer density distribution of the longer (shorter) brush grafted at the right wall ($z_{g2}=H$). The sum $\rho_{1}\left(z\right)+\rho_{2}\left(z\right)=\rho_{1}^{\left(1\right)}\left(z\right)+\rho_{1}^{\left(2\right)}\left(z\right)+\rho_{2}^{\left(1\right)}\left(z\right)+\rho_{2}^{\left(2\right)}\left(z\right)$ and the product $\rho_{1}\left(z\right)\rho_{2}\left(z\right)=\left(\rho_{1}^{\left(1\right)}\left(z\right)+\rho_{1}^{\left(2\right)}\left(z\right)\right)\left(\rho_{2}^{\left(1\right)}\left(z\right)+\rho_{2}^{\left(2\right)}\left(z\right)\right)$ of the two total profiles are also shown. As in the case of monodisperse semiflexible brushes, the increase in the chain stiffness results in a more pronounced extension of the chains away from the grafting wall, yielding a stronger overlap between the two brushes (see the discussion of Figure 8 below).

Considering next the PMF between the two equimolar bidisperse semiflexible brushes, Figure 7b shows the DFT results for W(z) for 5 different values of the chain stiffness parameter at the fixed grafting density $\sigma_{g}=0.0625$ and chain lengths $N_{1}=25$ and $N_{2}=16$. DFT results show that the strength of the brush-brush attraction decreases with increasing chain stiffness, similar to the monodisperse case. The minimum location of W(z) is largely insensitive to the value of the stiffness parameter κ.

The DFT results for both structural and energetic properties of equimolar bidisperse semiflexible brushes and their dependence on the chain stiffness are summarized in Figure 8, with the upper panel showing the overlap *P* and the lower panel presenting the (negative) PMF minimum −$W_{\min}$ as a function of the chain stiffness parameter. The grafting density is fixed at $\sigma_{g}=0.0625$ and grafted chain lengths are $N_{1}=25$ and $N_{2}=16$. As expected from the trends seen in Figure 7, *P* increases and −$W_{\min}$ decreases monotonically with κ, with the former reaching saturation around κ=10 Thus, the amount of overlap anticorrelates with the attraction strength between the two bidisperse brushes, similar to the monodisperse case (Figure 4).

To summarize the DFT results for bidisperse brushes, the overlap decreases monotonically with $\sigma_{g}$ and *N*, but increases with κ. On the other hand, the attraction strength decreases monotonically with increasing $\sigma_{g}$, *N*, and κ. Among these three control parameters, the grafting density has the strongest effect both on the overlap and the attraction strength between the two bidisperse brushes.

## 5. Conclusions

In this work we have applied the DFT method to study structural and energetic properties of two opposing solvent-free polymer brushes. The three control parameters that we have considered are the grafting density, the grafted chain length, and its stiffness parameter. In order to make a connection with the existing MD simulation data [26], both monodisperse and equimolar bidisperse brushes have been studied. Starting with monodisperse brushes, DFT results show that the monomer density profiles become more localized at the grafting wall, both with increasing *N* (at fixed $\sigma_{g}$) and with increasing $\sigma_{g}$ (at fixed *N*), in agreement with MD simulation results [26]. Concomitantly, the overlap between the two brushes diminishes, and the DFT results for the overlap parameter *P* are in good quantitative agreement with the corresponding MD data [26]. Increasing the grafted chain stiffness results in a more pronounced extension of the chains away from the grafting wall, producing a stronger overlap between the two brushes. The DFT results for the PMF between the two monodisperse rushes show that their attraction strength decreases with increasing *N* and κ, but increases with $\sigma_{g}$.

Moving next to bidisperse brushes, DFT again properly reproduces the trends seen in the simulated [26] density profiles. In particular, either increasing both chain lengths (at fixed $\sigma_{g}$) or increasing the grafting density (at fixed $N_{1}$, $N_{2}$) results in a more pronounced localization of the shorter chains at the grafting wall and the stretching of the longer chains away from it. In both cases, the overlap between the two brushes diminishes (with DFT and MD results for *P* in quantitative agreement) and their attraction becomes weaker. In terms of the two control parameters, the grafting density has a significantly stronger effect on both the overlap and the PMF well depth compared to the grafted chain lengths. The effect of the chain stiffness on the structure and energetics of the bidisperse brushes is similar to the monodisperse case, with the overlap increasing and the attraction strength decreasing for stiffer chains.

In the present work, we have focused exclusively on the *equilibrium* structural and energetic aspects of the two interacting brushes. In addition, it would be of interest to study various dynamic observables that were measured both in experiments [20] and in MD simulations [26]. This goal can be achieved either by developing a time-dependent DFT [63] or by combining the DFT framework with the mode-coupling theory [64]. Furthermore, the present work was limited to flat brushes, while it is equally important to study solvent-free polymer-grafted nanoparticles, whose core radius is similar to the grafted chain gyration radius [25]. These projects will be the subject of future research.

Another important issue in the field of polymer nanocomposites concerns their glass transition temperature [65]. For example, the proper functioning of devices based on shape memory materials may require a lower glass transition temperature, while temperature sensors might need a higher glass transition temperature. Accordingly, it would be of interest to apply DFT- and MCT-based methodology to study the effects of brush bidispersity and chain stiffness on the glass transition temperature of polymer nanocomposites.

## Data Availability

The data that support the findings of this study are available from the corresponding author upon reasonable request.
